# Treatment in canine epilepsy – a systematic review

**DOI:** 10.1186/s12917-014-0257-9

**Published:** 2014-10-22

**Authors:** Marios Charalambous, David Brodbelt, Holger A Volk

**Affiliations:** Department of Clinical Science and Services, Royal Veterinary College, Hawkshead Lane, Hatfield, Herts AL9 7TA UK; Department of Production and Population Health, Royal Veterinary College, Hawkshead Lane, Hatfield, Herts AL9 7TA UK

**Keywords:** Systematic review, Epilepsy, Antiepileptic drugs, Treatment, Canine

## Abstract

**Background:**

Various antiepileptic drugs (AEDs) are used for the management of canine idiopathic epilepsy (IE). Information on their clinical efficacy remains limited. A systematic review was designed to evaluate existing evidence for the effectiveness of AEDs for presumptive canine IE. Electronic searches of PubMed and CAB Direct were carried out without date or language restrictions. Conference proceedings were also searched. Peer-reviewed full-length studies describing objectively the efficacy of AEDs in dogs with IE were included. Studies were allocated in two groups, i.e. blinded randomized clinical trials (bRCTs), non-blinded randomized clinical trials (nbRCTs) and non-randomized clinical trials (NRCTs) (group A) and uncontrolled clinical trials (UCTs) and case series (group B). Individual studies were evaluated based on the quality of evidence (study design, study group sizes, subject enrolment quality and overall risk of bias) and the outcome measures reported (in particular the proportion of dogs with ≥50% reduction in seizure frequency).

**Results:**

Twenty-six studies, including two conference proceedings, reporting clinical outcomes of AEDs used for management of IE were identified. Heterogeneity of study designs and outcome measures made meta-analysis inappropriate. Only four bRCTs were identified in group A and were considered to offer higher quality of evidence among the studies. A good level of evidence supported the efficacy of oral phenobarbital and imepitoin and fair level of evidence supported the efficacy of oral potassium bromide and levetiracetam. For the remaining AEDs, favorable results were reported regarding their efficacy, but there was insufficient evidence to support their use due to lack of bRCTs.

**Conclusions:**

Oral phenobarbital and imepitoin in particular, as well as potassium bromide and levetiracetam are likely to be effective for the treatment of IE. However, variations in baseline characteristics of the dogs involved, significant differences between study designs and several potential sources of bias preclude definitive recommendations. There is a need for greater numbers of adequately sized bRCTs evaluating the efficacy of AEDs for IE.

## Background

Epilepsy is the most common chronic neurological disorder in dogs, with a formerly reported prevalence of between 0.5% and 5% in non-referral populations [1,2]. In a recent study, this prevalence was estimated to be 0.62% in a large UK primary care population [3]. Epilepsy is not one single disease process but can be elicited by multiple causes and, accordingly, can be classified as genetic (primary or idiopathic), structural and of unknown origin/etiology [4]. When chronic recurring seizures occur and no underlying abnormality is detected, epilepsy is classified typically as primary or idiopathic epilepsy [1]. However, idiopathic epilepsy could imply a potential genetic background and in veterinary medicine the terms idiopathic or primary are generally used for any epilepsy of unidentified etiology even if no genetic or familial causes are suspected [5]. In this study the term idiopathic epilepsy (IE) will be used for all the cases of unidentified etiology, including cases with a suspected genetic background.

Various antiepileptic drugs (AEDs) are used for the management of IE in dogs. Clinical information on the grounds of their efficacy remains limited, with most evidence derived from non-blinded non-randomized uncontrolled trials and case series [6]. In addition, many of these previous reports do not use an objective measurement of efficacy, e.g. a% reduction in seizure frequency in a proportion of dogs of a study population after a specific period of treatment; instead they are based on subjective observations, e.g. ‘improvement in seizure control’ or ‘change in seizure frequency’.

To the authors’ knowledge, this is the first systematic review in veterinary medicine which evaluates studies that describe the efficacy of AEDs used for the management of IE, based on objective criteria. The review evaluated clinical trials and case series with measurement of AED efficacy.

## Methods

### Search strategy

The literature search aimed to identify all studies evaluating the clinical effectiveness of an AED in dogs with presumptive IE. Specifically, studies were evaluated based on the inclusion criteria below:

- Criterion 1-Type of study: Peer-reviewed studies in English (or translated). Clinical trials and case series were included.
- Criterion 2-Case definition: Dogs with IE, with a reported unremarkable (i.e. absence of neurological deficits) inter-ictal period and age range of 6 months to 7 years old and diagnosed after investigation for exclusion of any underlying cause. Brain magnetic resonance imaging (MRI), computer tomography (CT) and/or cerebrospinal fluid (CSF) analysis confirmation were preferable but not essential. Dogs with confirmed or suspected extra-cranial disease, i.e. metabolic or intracranial pathology, e.g. brain tumor, were excluded. Dogs manifesting generalized, simple/complex focal with or without secondary generalization were included.
- Criterion 3-Treatment: Dogs treated chronically with any AED available were included. Doses of AEDs, frequency of drug administration and treatment period were required to be reported. Dogs treated with methods other than pharmacological intervention, e.g. homoeopathy methods, surgery, food trials, nerve stimulation, were excluded.
- Criterion 4-Outcome: Studies had to include (or provide adequate data for calculations of) specific outcome parameters such as alterations in seizure frequency, expressed as percentage or other numeric values for an identified length of time after the AED initiation. Assessment of the response to treatment should have been performed by the veterinarian or owner. The time needed for the AED to result in a clinically significant reduction of seizures should have been reported.

Search strategies included use of electronic search engines for publication databases, searching of reference lists of published papers and proceedings of relevant scientific conferences. Electronic databases used were Pub Med (www.ncbi.nlm.nih.gov/PubMed) and CAB Abstracts (www.cabdirect.org). Final electronic searches were carried out on 10 August 2014 by the primary author, with no date or language restrictions. The search terms used in both search engines were as follows: (dog OR dogs OR canine) AND [(phenobarbital OR primidone OR potassium bromide OR bromide OR nimodipine OR zonisamide OR ELB138 OR imepitoin OR levetiracetam OR verapamil OR gabapentin OR gaba OR topiramate OR felbamate OR pregabalin) OR [(treatment OR management) AND (epilepsy OR seizures)] OR anti-seizuring OR anti-epileptic OR AED]. Searching for articles from the reference lists of publications and searching major veterinary neurology conference meeting proceedings from 1980 to 2013 was carried out by the primary author. Conferences meetings searched were as follows: Annual Congresses of the European Society and College of Veterinary Neurology (ESVN ⁄ ECVN) and the American College of Veterinary Internal Medicine (ACVIM). Other conference meetings were searched only if the reference list of identified publications indicated this. All items returned by the search engines, hand searches and correspondence were recorded and entered into the screening process.

### Study selection

Restrictions based on publication date or language were not imposed. Studies written in non-English language were assessed initially based on an English translation (Google Translate software) and then verified by a veterinarian fluent in the language of publication.

A two-stage process of screening was used by the first author. Firstly, studies of relevance to the systematic review questions were identified (stage 1) and, secondly, studies likely to provide evidence of the highest available quality and sufficient detail for assessing the outcome measures and methodology were selected (stage 2). Stage 1 of the screening process identified from the total search results any studies that: (a) fulfilled inclusion criterion 1 and (b) reported findings related to the effects of in-vivo treatment in IE. Stage 1 assessment evaluated the retrieved papers’ titles and abstracts only. At stage 2, papers were selected for full data extraction according to the inclusion criteria 2, 3 and 4 and were evaluated in detail on the grounds of the quality of evidence and treatment outcomes.

### Assessment of quality of evidence

Study design was determined in each trial selected for review; blinded randomized clinical trials (bRCTs) were considered most likely to produce higher quality of evidence, followed by non-blinded randomized clinical trials (nbRCTs), then non-randomized clinical trials (NRCT), uncontrolled clinical trials (UCTs) and lastly case series [7]. Accordingly, the studies were allocated based on their design to one of two groups, i.e. bRCTs, nbRCTs and NRCTs (first group) and UCTs and case series (second group). In addition, a three-part system of evidence quality assessment to indicate the strengths and weaknesses of each study in each group was used [8]: (a) study group sizes, (b) subject enrolment quality and (c) overall risk of bias based on methodological quality, in order to provide an indicator of confidence associated with the findings of each study. For instance, bRCTs with large group sizes, clear inclusion criteria and diagnostic investigations that included clinical signs and thorough test results consistent with the diagnosis of IE, describing outcomes specific for IE and low overall risk of bias were considered to provide the highest available quality of evidence. The studies in group A were considered to provide higher quality of evidence than the studies in group B. For the studies selected in stage 2, summaries of the assessment of quality of evidence are provided in Table 1.

**Table 1:** **Summaries of the quality of evidence of each study**

	**Study Groups**	**Study design**	**Overall ‘risk of bias**	**Disease definitions (characterization)**	**Study groups size**
**1. Boothe et al.** [11]	A	bRCTs	Low/Moderate	Poorly	Moderate
**2. EMEA pseudo-trial** [13]				Unclear	Good
**3. Tipold et al.** [14]				Poorly	Good
**4. Muñana et al.** [12]				Poorly	Moderate
**5. Schwartz-Porsche et al.** [15]		nbRCT	Moderate/High	Unclear	Moderate
**6. Chung et al.** [24]	B	UCTs	Moderate/High	Well	Small
**7. Cunningham et al.** [29]				Well	Small
**8. Dewey et al.** [18]				Fairly	Very small
**9. Dewey et al.** [19]				Fairly	Small
**10. Kiviranta** [17]				Fairly	Small
**11. Platt et al.** [22]				Poorly	Small
**12. Pearce** [32]				Fairly	Small
**13. Volk et al.** [16]				Well	Small
**14. Schwartz-Porsche** [28]				Well	Small
**15. Schwartz-Porsche et al.** [30]				Poorly	Moderate
**16. Steinberg** [23]				Unclear	Small
**17. Rieck et al.** [27]			High	Fairly	Small
**18. Govendir et al.** [21]				Poorly	Small
**19. Von Klopmann et al.** [20]				Fairly	Small
**20. Löscher et al.** [26]				Fairly	Small
**21. Morton et al.** [31]				Unclear	Small
**22. Nafe** [25]				Fairly	Moderate
**23. Heynold** [36]		Retrospective case series studies		Fairly	Moderate
**24. Podell et al.** [33]				Fairly	Moderate
**25. Ruehlmann et al.** [35]			Moderate/High	Fairly	Very small
**26. Trepanier et al.** [34]				Unclear	Good
**Löscher et al.** [26] **(retrospective part)**			As a part of trials	Fairly	Moderate
**Rieck et al.** [27] **(retrospective part)**				Fairly	Moderate
**Volk et al.** [16] **(retrospective part)**				Well	Very Small

bRCTs, blinded randomized clinical trials; CTs, clinical trials; nbRCTs, non-blinded randomized clinical trials; NRCTs, non-randomized clinical trials.

### Study group sizes

This characteristic was categorized for each study using the following system [8]: (a) >50 subjects per group (‘good’ number of subjects), (b) 20–50 subjects per group (‘moderate’ number), (c) 10– 19 subjects per group (‘small’ number) and (d) <10 subjects per group (‘very small’ number).

### Assessment of subject enrolment quality

Data on investigations to reach the diagnosis of IE were retrieved to evaluate the quality of subject enrolment in each study as ‘well characterized’, ‘fairly characterized’, ‘poorly characterized’ or ‘unclear’: Well characterized diagnoses were defined as diagnostic investigations that included clinical signs and thorough test results consistent with the diagnosis of IE; specifically, the signalment, the absence of neurological deficits between the ictal phases, unremarkable blood tests and imaging results (including brain MRI and/or CT) and/or normal cerebrospinal fluid (CSF) analysis for all cases of the study. Fairly characterized, used for intermediate situations, were defined as where diagnosis was based on signalment, clinical examination and basic diagnostic investigation (i.e. blood tests) with some cases having had advanced brain imaging and/or CSF analysis. Poorly characterized were studies where diagnosis was based on signalment, clinical examination and basic diagnostic investigation (i.e. blood tests) only. Unclear related to reports where the approach to diagnosis of IE was not clearly stated (e.g. when clinical signs were not stated and insufficient or no details of diagnostic tests were provided or when dogs with IE were included without reporting details on diagnostic investigation).

### Assessment of methodological quality

Using the criteria for judging risk of bias in the Cochrane ‘risk of bias’ assessment tool [9], each of the following study components was categorized as presenting a ‘high’, ‘moderate’ or ‘unclear’ or ‘low’ risk of introducing bias to the study findings: random sequence generation, allocation concealment, blinding of participants, personnel and outcome assessors, completeness of outcome data, selective reporting of outcomes and other sources of bias. The overall risk of bias for each study was estimated by combining the risk of bias from all the components. Each of the seven components was assigned a numerical score and summed to form a total score. This translated to an overall estimated risk of bias for each study. Further details are presented in Table 2.

**Table 2:** **Numerical scoring system used to allocate a score-based grade for the overall risk of bias in each of the twenty-six reviewed studies**

**Studies**	**Randomisation sequence generation**	**Allocation concealment**	**Blinding of participants and personnel**	**Blinding of outcome assessment**	**Incomplete outcome data**	**Selective reporting**	**Other bias**	**Overall ‘risk of bias' category**
Boothe et al. [11]	1	1	1	2	2	2	2	Low/moderate (11)
							Primary investigator could potentially influence the treatment.	
Chung et al. [24]	3	3	3	3	1	2	2	Moderate/high (17)
							Research support but unclear if it was financial.	
Cunningham et al. [29]	3	3	3	3	2	2	2	Moderate/high (18)
							Conference abstract	
Dewey et al. [18]	3	3	3	3	2	2	2	Moderate/high (18)
							Less than 6 months study duration.	
Dewey et al. [19]	3	3	3	3	2	2	1	Moderate/high (17)
EMEA pseudo-trial [13]	1	2	1	3	1	2	2	Low/moderate (12)
							The follow up assessment of efficacy was not blinded. Different drug formulations were used compared to the final formulation.	
Govendir et al. [21]	3	3	3	3	2	2	3	High (19)
							A few cases were treated by the referring vets. The study had financial support. Less than 6 months study duration.	
Heynold et al. [36]	3	3	3	3	2	2	3	High (19)
							The study had financial support but unclear if it influenced the results. Less than 6 months study duration.	
Kiviranta [17]	3	3	3	3	2	2	1	Moderate/high (17)
Löscher et al. [26]	3	3	3	3	2	2	3	High (19)
							Part of the study was retrospective	
Morton et al. [31]	3	3	3	3	2	2	3	High (19)
							A few cases were treated by the referring vets. The study had financial support but unclear if it influenced the results.	
Muñana et al. [12]	1	1	1	1	2	2	2	Low/moderate (10)
							The study had financial support but unclear if it influenced the results.	
Nafe [25]	3	3	3	3	2	2	3	High (19)
							Less than 6 months study duration.	
Pearce [32]	3	3	3	3	2	2	2	Moderate/high (18)
Platt et al. [22]	3	3	3	3	1	2	3	Moderate/high (18)
							Less than 6 months study duration.	
Podell et al. [33]	3	3	3	3	2	2	3	High (19)
							Retrospective nature of study.	
Rieck et al. [27]	3	3	3	3	2	2	3	High (19)
							Part of the study was retrospective	
Ruehlmann et al. [35]	3	3	3	3	1	2	3	Moderate/high (18)
							Part of the study was retrospective. No clarification of statistical analysis	
Schwartz-Porsche [28]	3	3	3	3	2	2	2	Moderate/high (18)
Schwartz-Porsche et al. [15]	2	2	3	3	2	2	2	Moderate/high (16)
							The study had research support but unclear if it influenced the results. No clarification of statistical analysis	
Schwartz-Porsche et al. [30]	3	3	3	3	2	2	1	Moderate/high (17)
Steinberg [23]	3	3	3	3	2	2	2	Moderate/high (18)
							Conference abstract	
Tipold et al. [14]	1	2	1	1	1	2	3	Low/moderate (11)
							Statistical analysis was conducted before unblindingonly on the per-protocol population and not on the intent-to-treat population. A high and unbalanced population of animals was excluded. The reasons for exclusion were in many cases treatmet-related (post-randomization bias). Conflict of interest about imepitoin reported.	
Trepanier et al. [34]	3	3	3	3	2	2	2	Moderate/high (18)
							Some samples were submitted by the referring vets.	
Volk et al. [16]	3	3	3	3	1	2	3	Moderate/high (18)
							The study had financial support but unclear if it influenced the results. Part of the study was retrospective	
Von Klopmann et al. [20]	3	3	3	3	2	2	3	High (19)
							Less than 6 months study duration.	

Each aspect the risk of bias was categorised as ‘high’, ‘moderate’, ‘low’ or ‘unclear’. These categories were assigned a numerical score as follows: High risk of bias =3, moderate or unclear risk of bias =2, low risk of bias =1. Within each study these seven scores were summed to form a total score. This score translates to an overall estimated risk of bias associated with the findings of the study in question, as follows: Score 19–21 = overall high risk of bias, score 16 – 18 = overall moderate/high risk of bias, score 13 – 15 = overall moderate risk of bias, score 10 – 12 = overall low/moderate risk of bias, score 7 – 9 = overall low risk of bias.

### Assessment of outcome measures

The outcome measure of this review was the evaluation of the treatment efficacy of AED(s) administered in dogs with IE. The outcome measure was assessed by the level of evidence for/against supporting the use of an AED based on studies’ results as well as the proportion of dogs in the study population that had a reduction in seizure frequency. The latter was reported as the percentage seizure frequency reduction from baseline or was calculated by the authors where sufficient data were available, i.e. where both sample size and seizure frequency reductions were reported. Dogs with ≥50% reduction in seizure frequency were considered as successfully treated cases. Dogs with ≥0% to <50% reduction in seizure fequency were considered as cases having an inadequate response to AED treatment. The proportion of study dogs successfully treated as defined above and its 95% confidence interval (95% CI) were calculated by standard methods [10]. The 95% CI of dogs successfully treated was used as a further indicator of treatment efficacy. Precisely, if the 95% CI of dogs successfully treated was greater than 50% (95% CI of proportion of successfully treated dogs ≥0.50), then it was considered that the majority of the dogs were successfully managed.

Statements of efficacy of individual AED(s) from each study were reported separately but the overall evidence for/against recommending the use of an AED was allocated according to the following system and based on the proportion of dogs that were successfully treated [8]: ‘good’ evidence ‘for’ recommending use of the drug, when at least one bRCT strongly supported the efficacy of the drug used for IE; ‘fair’ evidence ‘for’ recommending use of the drug, when at least one bRCT fairly supported the efficacy of the drug used for IE; ‘insufficient’ evidence ‘for ⁄ against’ recommending the use of the drug, when studies were supportive (or not) of the use of an AED but bRCTs were not available; ‘fair’ evidence ‘against’ recommending the use of the drug, when at least one bRCT fairly supported the lack of efficacy; ‘good’ evidence ‘against’ recommending the use of the drug, when at least one bRCT strongly supported the lack of efficacy of the medication tested; and ‘unclear’ evidence, when bRCTs presented conflicting results (i.e. studies ‘for’ and studies ‘against’ recommending the use of drug) occurred together for the same AED.

A meta-analysis to identify similar patterns and sources of disagreement among study results concerning the efficacy of the AEDs was considered in the study protocol dependent on the evidence identified.

## Results

### Description of studies

By 10 August 2014, the search strategy had identified a total of 156 unique citations; 142 from the electronic searches of PubMed and CAB Abstracts and manual searches from the publications’ reference lists, and 14 from manual searching of major conference proceedings. One hundred four published items fulfilled stage 1 screening criteria. Of these, 26 individual studies (published between 1981 and 2014) also fulfilled stage 2 selection criteria and were thus selected for review.

Five and 21 studies were allocated in group A and B, respectively. Study designs represented were four bRCT [11-14] and one nbRCT [15] in group A and 17 UCTs [16-32] and four retrospective case series [33-36] in group B. One study was published in German [28]. Summaries of the design for each study are provided in Table 1.

Overall, the 26 selected studies reported 11 AEDs. All AEDs were orally administered. Within each study one or more AEDs were evaluated as a monotherapy and/or adjunct to other AEDs.

### Epilepsy characterization

According to the described grading system for subject enrolment quality, four studies [16,24,28,29] enrolled treatment groups of well characterized IE, 11 studies [17-20,25-27,32,33,35,36] enrolled treatment groups of fairly characterized IE and six studies [11,12,14,21,22,30] enrolled treatment groups of poorly characterized IE. In five studies [13,15,23,31,34], the diagnostic procedures for enrollment of cases with IE were unclear (Table 1).

### Study group sizes

All 26 studies reported the total number of dogs evaluated (range 6–127 dogs; median 16 dogs; mean 27 dogs). Three of the selected studies evaluated a good number of dogs [13,14,34]. Five trials [11,12,15,25,30] and two case series [33,36] evaluated groups with a moderate number of dogs, 14 trials [16,17,19-24,26-29,31,32] evaluated groups with a small number of dogs and one trial [18] and one case series [35] evaluated groups with a very small number of dogs. Summaries of the group size for each study are provided in Table 1.

### Signalment and baseline characteristics of study subjects

Baseline characteristics (such as breed, age and sex) of total enrolled dogs were reported to some extent for all 26 studies. Clear presentation of statistical comparison of intervention groups with respect to signalment and baseline disease characteristics pretreatment was not commonly encountered.

One study [36] described results specifically for one breed (Labrador retrievers). In all other trials reporting baseline data, the recruited dogs represented multiple breeds, both sexes and a wide range of ages at study entry (median 5, mean 4, range 0.5-7). In the majority of the studies more males were affected compared to females. Major affected breeds were crossed-breeds and pure breeds such as Labrador and Golden retrievers followed by German shepherd dogs, beagles, boxers and poodles.

### Methodological quality of included studies

Based on the criteria outlined in the review protocol, in group A four studies [11-14] and one study [15] were considered to be at low/moderate and moderate/high overall risk of bias, respectively. All the remaining 21 studies in group B were considered as to be at overall moderate/high risk of bias, apart from eight studies which were considered to be at overall high risk of bias [20,21,25-27,31,33,36]. Summaries of the risk of bias for each study are provided in Table 2.

### Method of randomization and allocation concealment

In group A, all five studies [11-15] used randomization to allocate the dogs and were considered to provide a low risk of bias. Three studies [13-15] did not offer enough detail to confirm that allocation concealment was used, whilst two studies stated that randomization was concealed. One study [11] assigned by random blocking (random allocation to blocks of 10) and the other one [12] used a computer-generated list of random numbers. The studies of group B did not use randomization.

### Blinding of outcome assessment

In group A, blinding was clearly described in three studies [11-14] which were also considered to be at low risk. In these three studies, blinding was applied to all participants, personnel and outcome assessment. In one of them [11] all but the primary investigator were blinded and in another one [13] the final outcome assessment was not blinded. One study of group A [15] was not blinded; so it was considered to be at high risk. For the studies of group B, blinding was not used.

### Incomplete outcome data

Five studies presented outcome data from all enrolled dogs in the treatment group to which they were originally allocated and there were no losses between enrolment and evaluation [13,16,22,24,35]. The same studies were considered to be at low risk. In two studies, it was unclear whether all dogs completed the study, as inadequate information was provided [23,29]. Across the remaining studies, there were dogs which were euthanized or excluded due to poor seizure control, side effects, at the owner’s request or for unidentified reasons; thus there were losses between the initial inclusion population and the final number of the dogs.

### Selective reporting

It was difficult to assess selective reporting as study protocols were not sought beyond the information published; therefore no studies from any group were judged to be free from bias for selective reporting.

### Acknowledgment of other sources of bias

Seven studies reported financial support [12,15,16,21,24,31,36] but it was judged unclear whether this biased the results. One study [17] clearly mentioned that there was no financial support, while the remaining 18 studies [11,13,14,18-20,22,23,25-30,32-35] failed to report financial support.

Six studies [18,20-22,25,36] were considered to be of inadequate study duration (less than six months). In two studies [15,35] the statistical analysis was not clarified. In one study [14], statistical analysis was conducted before unblinding only on the per-protocol population and not on the intent-to-treat population and also post-randomization bias occurred (treatment-related exclusions of dogs). Two studies [23,29] were conference abstracts, thus no further information could be retrieved. One dog in one study [32] and two dogs in two studies [15,28] were diagnosed with symptomatic epilepsy (i.e. a cause was identified); which could potentially affect the final results on AED efficacy. One study [28] reported the term “drug refractory cases” but without providing further definition. Conflict of interest was clearly stated in one study [14].

### Efficacy of AEDs

Details of seizure frequency reduction/response after the initiation of treatment, pre- and post- treatment seizure frequency, doses of AED(s) and period of treatment for each study are provided in Tables 3, 4, 5, 6, 7, 8 and 9. Also, the overall evidence for/against recommending the use of each AED as well as the 95% CI of the proportion of successfully treated cases for each study are presented below and in Tables 3, 4, 5, 6, 7, 8 and 9.

**Table 3:** **Details of numbers of dogs, pre- and post- treatment seizure frequency, period of treatment, doses of AED(s), seizure frequency reduction/response after the initiation of treatment and efficacy statements for each study**

**References**	**Boothe et al.** [11]	**Schwartz-Porsche et al.** [15]	**Löscher et al.** [26] **Rieck et al.** [27]	**Morton et al.** [31]	**Heynold et al.** [36]	**Tipold et al.** [14]
**AED evaluated**				**Phenobarbital**		
**2** ^**nd**^ **AED**	-	-	-	-	-	-
**3** ^**rd**^ **AED**	-	-	-	-	-	-
**4** ^**th**^ **AED**	-	-	-	-	-	-
**No of dogs**	20	15	44	7	37	After exclusion: 88
						Before exclusion: 102
**Period of treatment (months)**	approx. 6	mean 15; range 7.3-32	mean 5.9 +/−0.4	Unclear	mean 50.4; range 8-18	5
**Dose of AED(s) (mg/kg)**	mean 4.11; range 3.9-4.9 PO BID	Range 5–17 PO SID	mean 6; range 4–13 PO SID	median 180; mean 283; range 60–900 PO SID	mean 2.5 PO BID	2-6 PO BID
**Pre-treatment SF (seizures/month)**	mean 4.4 +/− 6.3 (recorded over a period of at least 6 weeks)	NA	mean 1.71 (recorded over a period of 9 m)	median 12; mean 14.3; range 4–28 (period not reported)	mean 8 seizures in total (period not reported)	2.4
**Post-treatment SF (seizures/month)**	mean 0.4 +/− 0.9	NA	mean 0.59	median 1; mean 1; range 0-4	mean 0.9	1.1
**No of dogs that were failures**	-	3/15 (20%)	12/44 (27%)	1/7 (14%)	10/37 (27%)	-
**No of dogs with >0% - <50% reduction in SF**	2/20 (10%)	-	-	3/7 (43%)	-	-
**No of dogs with ≥50% - <100% reduction in SF**	1/20 (5%)	6/15 (40%)	28/44 (64%)	-	16/37 (43%)	After exclusion: 22/88 (25%)
						Before exclusion: 25/102 (24%)
**No of dogs with 100% reduction in SF**	17/20 (85%)	6/15 (40%)	9/44 (20%)	3/7 (43%)	11/37 (30%)	After exclusion: 51/88 (51%)
						Before exclusion: 56/102 (55%)
**No of dogs with >30% reduction in SF**	-	12/15 (80%)	37/44 (84%)	4/7 (57%)	27/37 (73%)	-
**95% CI successfully treated cases**	77% - 100%	60% - 100%	73% - 95%	6% - 80%	59% - 87%	After exclusion: 75% - 91%
						Before exclusion: 71% - 87%
**Overall evidence for/against recommending the use of an AED**	Good evidence for recommending the use of phenobarbital as a monotherapy AED.					

AED(s), anti-epileptic drug(s); BID, bis in die (twice daily); Chloraz, Chlorazepate; CSF, cerebrospinal fluid; CL, confidence level; Diaz, Diazepam; Gaba, Gabapentin; IE, idiopathic epilepsy; LEV, Levetiracetam; m, month(s); NA, Not Available; PB, phenobarbital; PBr, potassium bromide; PO, per os; SF, seizure frequency; SID, semel in die (once daily); TID, ter in die (three times daily); TPM, Topiramate; w, week(s).

The evaluated AED is the one whose efficacy was assessed. This AED was administered and evaluated either as a monotherapy agent in previously untreated animals or as a monotherapy after an alteration in its dose or as a new AED in previously treated, with other AED(s), animals.

**Table 4:** **Details of numbers of dogs, pre- and post- treatment seizure frequency, period of treatment, doses of AED(s), seizure frequency reduction/response after the initiation of treatment and efficacy statements for each study**

**References**	**Tipold et al.** [14]	**EMEA pseudo-placebo** [13]	**Löscher et al.** [26] **Rieck et al.** [27]	**Löscher et al.** [26] **Rieck et al.** [27]
**AED evaluated**		**Imepitoin**		
**2** ^**nd**^ **AED**	-	-	-	PB (11 dogs) or Primidone (6 dogs)
**3** ^**rd**^ **AED**	-	-	-	-
**4** ^**th**^ **AED**	-	-	-	-
**No of dogs**	After exclusion: 64	First part: 127	12	17
	Before exclusion: 93	Second part (follow up): 100 (from the 127)		
**Period of treatment (months)**	5	6	mean, 7.7 ± 0.7	mean, 5.6 ± 0.7
**Dose of AED(s) (mg/kg)**	10-30 PO BID	High dose group: 30 PO BID	5 PO BID for 1 week and then increased to 10–30 PO BID	Imepitoin: mean, 7.7 ± 0.7;
		Low dose: 1 PO BID [during the follow up all the 100 (53 from the previous high dose group and 47 from the low-dose) dogs were treated with the high dose only]		PB: 6–23 PO SID; Primidone: 25–53 PO SID
**Pre-treatment SF (seizures/month)**	2.3 (recorded over a period of 1.5 m)	High dose group: mean, 2.9	median, 1.6 (recorded over a period of approx. 9 m)	median, 1.9 (recorded over a period of mean 1.6 years)
		Low dose group: mean, 2		
**Post-treatment SF (seizures/month)**	1.1	High dose group: mean, 2.2	median, 0.72	median, 2
		Low dose group: mean, 1.8		
		(For the follow up study: NA)		
**No of dogs that were failures**	-	Unclear	3/12 (25%)	6/17 (35%)
**No of dogs with >0% - <50% reduction in SF**	-	Unclear	4/12 (33%)	4/17 (24%)
**No of dogs with ≥50% - <100% reduction in SF**	After exclusion: 18/64 (28%)	Unclear	4/12 (33%)	6/17 (35%)
	Before exclusion: 22/93 (24%)			
**No of dogs with 100% reduction in SF**	After exclusion: 30/64 (47%)	First Part: High dose group: 44/127 (35%); Low dose: 6/127 (5%)	1/12 (8%)	1/17 (6%)
	Before exclusion:31/93 (33%)	Follow-up: High-high dose group: 19/53 (35%) and Low-high dose group: 24/47 (50%)		
**No of dogs with >30% reduction in SF**	-	As above	9/12 (75%)	11/17 (65%)
**95% CI successfully treated cases**	After exclusion: 64% - 86%	Follow-up: 25-46% and 36-63% (but only for the seizure free dogs)	13% - 69%	18% - 64%
	Before exclusion: 47% - 67%			
**Overall evidence for/against recommending the use of an AED**	Good evidence for recommending the use of imepitoin as a monotherapy AED.			
	Insufficient evidence for recommending the use of imepitoin as an adjunct AED.			

AED(s), anti-epileptic drug(s); BID, bis in die (twice daily); Chloraz, Chlorazepate; CSF, cerebrospinal fluid; CL, confidence level; Diaz, Diazepam; Gaba, Gabapentin; IE, idiopathic epilepsy; LEV, Levetiracetam; m, month(s); NA, Not Available; PB, phenobarbital; PBr, potassium bromide; PO, per os; SF, seizure frequency; SID, semel in die (once daily); TID, ter in die (three times daily); TPM, Topiramate; w, week(s).

**Table 5:** **Details of numbers of dogs, pre- and post- treatment seizure frequency, period of treatment, doses of AED(s), seizure frequency reduction/response after the initiation of treatment and efficacy statements for each study**

**References**	**Boothe et al.** [11]	**Schwartz-Porsche et al.** [28]	**Löscher et al.** [26] **Rieck et al.** [27]	**Trepanier et al.** [34]	**Podell et al.** [33]	**Pearce** [32]
**AED evaluated**	**Potassium bromide**					
**2** ^**nd**^ **AED**	-	PB (15 dogs) or Primidone (4 dogs)	PB (8 dogs) or Primidone (4 dogs)	PB or Primidone	PB (23 dogs)	PB (10 dogs)
**3** ^**rd**^ **AED**	-	-	-	-	-	-
**4** ^**th**^ **AED**	-	-	-	-	-	-
**No of dogs**	23	19	44	122	37	10
**Period of treatment (months)**	Approx. 6	mean, 21; range, 7-61	mean, 7.3 ± 0.6	mean, 14.2 +/− 4.7	mean, 15; range, 4-33	median, 7; mean 7.8
**Dose of AED(s) (mg/kg)**	mean, 30.6; range, 26–35 PO BID	PBr: 17–58 PO SID;	PBr: 40–60 PO SID;	Doses were NA but adjusted according to the therapeutic serum levels and clinical response	PBr: mean, 20.75; range, 13–40 PO BID; PB: NA	PBr: 22 PO SID (dose increases occurred);
		PB and Primidone: NA but kept at maximum therapeutic doses	PB: 6–17 PO SID;			PB: median, 3.3; mean, 3.8 PO BID (dose was reduced by a mean of 50% in 7/10 dogs during the PBr treatment)
			Primidone: 50–70 PO SID			
**Pre-treatment SF (seizures/month)**	mean, 5.4 +/− 9.7 (recorded over a period of at least 6 weeks)	NA (but recorded over period of mean, 31; range, 8–79 m)	median, 3 (recorded over a period of mean 1.7 years)	NA	mean, 14.1 +/− 11.6 (recorded over a period of 0–12 m)	median, 27; mean 25; range 3–45 (recorded over a period of 5–72 m)
**Post-treatment SF (seizures/month)**	mean, 1.2 +/− 2.4	NA	median, 1.9	-	mean, 6.6 +/− 5.7	NA
**No of dogs that were failures**	3/23 (13%)	6/19 (32%)	5/12 (42%)	-	-	3/10 (30%)
**No of dogs with >0% - <50% reduction in SF**	1/23 (4%)	2/19 (10%)	2/12 (16%)	-	6/23 (26%)	-
**No of dogs with ≥50% - <100% reduction in SF**	5/23 (22%)	7/19 (37%)	5/12 (42%)	88/122 (72%)	11/23 (48%)	3/10 (30%)
**No of dogs with 100% reduction in SF**	12/23 (52%)	4/19 (21%)	-	-	6/23 (26%)	4/10 (40%)
**No of dogs with >30% reduction in SF**	-	17/19 (89%)	7/12 (32%)	88/122 (72%)	23/23 (100%)	7/10 (70%)
**95% CI successfully treated cases**	57% - 91%	36% - 80%	27% - 57%	64% - 80%	60% - 88%	42% - 98%
**Overall evidence for/against recommending the use of an AED**	Fair evidence for recommending the use of potassium bromide as a monotherapy AED.					
	Insufficient evidence for recommending the use of potassium bromide as an adjunct AED.					

AED(s), anti-epileptic drug(s); BID, bis in die (twice daily); Chloraz, Chlorazepate; CSF, cerebrospinal fluid; CL, confidence level; Diaz, Diazepam; Gaba, Gabapentin; IE, idiopathic epilepsy; LEV, Levetiracetam; m, month(s); NA, Not Available; PB, phenobarbital; PBr, potassium bromide; PO, per os; SF, seizure frequency; SID, semel in die (once daily); TID, ter in die (three times daily); TPM, Topiramate; w, week(s).

**Table 6:** **Details of numbers of dogs, pre- and post- treatment seizure frequency, period of treatment, doses of AED(s), seizure frequency reduction/response after the initiation of treatment and efficacy statements for each study**

**References**	**Volk et al.** [16]	**Volk et al.** [16] **(retrospective)**	**Steinberg M** [23]	**Muñana et al.** [12]	**von Klopmann et al.** [20]	**Dewey et al.** [19]	**Chung et al.** [24]
**AED evaluated**	**Levetiracetam**				**Zonisamide**		
**2** ^**nd**^ **AED**	PB (14 dogs)	PB (8 dogs)	PB (15 dogs)	PB (33 dogs)	PB (11 dogs)	PB (33 dogs)	-
**3** ^**rd**^ **AED**	PBr (14 dogs)	PBr (8 dogs)	PBr (15 dogs)	PBr (29 dogs)	PBr (4 dogs)	PBr (29 dogs)	-
**4** ^**th**^ **AED**	-	-	-	Gapa (2 dogs)	-	Felb (1 dog) or Gaba (1 dog) or Cloraz (1 dog)	-
**No of dogs**	14	8	15	22	11	12	10
**Period of treatment (months)**	2-6 or more	Approx. 2-3	median, 38; range, 13.8-95.5	9 (during the 5th m no AED was administered)	range, 4-17	mean, 8; median, 9; range, 2 -18	median, 12; mean, 11.2
**Dose of AED(s) (mg/kg)**	LEV: 10 for 2 m, 20 for further 2 m, 10–20 until 6 m and then 10–20 long-term PO TID; PB and PBr: NA but were within normal reference values	LEV: median, 22.15; mean, 21.7; range, 10–32.8 PO TID; PB and PBr: NA but were within normal reference values	LEV: range, 7.1-23.8 PO TID; PB and PBr: NA	lEV: median, 20.6; range, 17–23.1 PO TID; PB: median, 8.7; range, 2.9-17.2; PBr: median, 39.1; range, 13.6-133.3 PO SID)	Zonisamide: mean, 8.9; range, 5–11. PO BID; other AED doses were NA but continued unchanged or reduced	Zonisamide: mean, 8.9; range, 5–11. PO BID; other AEDs doses were NA but reduced or eliminated in 9/12 dogs	median, 9.5; mean, 8.65; range, 2.5-12 PO BID
**Pre-treatment SF (seizures/month)**	median, 7.25; mean 8.2 (recorded over a period of 2 m)	median, 8; mean 9.7 +/− 7.6; range 1–25 (period not recorded)	mean, 4.3 (recorded over a period of median 17 m)	median, 8.4+/−10; mean, 7.6 ± 7.6 (recorded over a period of 2 m)	median 6.5; range 1–72 (over a period of 4 m)	median 19.8; mean 33 (recorded over a period of 2.5 to 82 m)	median, 3; mean, 4.4; range, 2–10 (period not recorded)
**Post-treatment SF (seizures/month)**	4 m: median 3.5; mean 3.7. 6 m: median 4.25; mean 4.8	median, 0; mean 3.9 +/−6; range 0–15.5	mean, 1.96	mean, 4.4+/−5.2	median, 1.63; range, 0-9	mean, 1.8; median 3	median, 1.5; mean, 2.5; range, 0-10
**No of dogs that were failures**	4 m: 2/14 (15%) 6 m: 2/11 (18%)	-	-	-	-	5/12 (42%)	4/10 (40%)
**No of dogs with >0% - <50% reduction in SF**	4 m: 3/14 (21%) 6 m: 1/11 (9%)	-	-	-	2/10 (20%)	-	-
**No of dogs with ≥50% - <100% reduction in SF**	4 m: 6/14 (43%) 6 m: 7/11 (64%)	3/8 (37.5%)	15/15 (100%)	12/22 (56%)	6/10 (60%)	5/12 (42%)	2/10 (20%)
**No of dogs with 100% reduction in SF**	4 m: 3/14 (21%) 6 m: 1/11 (9%)	5/8 (62.5%)	-	4/22 (17%)	2/10 (20%)	2/12 (16%)	4/10 (40%)
**No of dogs with >30% reduction in SF**	4 m: 11/14 (79%) 6 m: 8/11 (73%)	5/8 (62.5%)	15/15 (100%)	-	8/10 (80%)	7/12 (58%)	6/10 (60%)
**95% CI successfully treated cases**	4 m: 39% - 89%	100%	100%	54% - 92%	56% - 100%	30% - 86%	30% - 90%
	6 m: 50% - 96%						
**Overall evidence for/against recommending the use of an AED**	Fair evidence for recommending the use of levetiracetamas an an adjunct AED.				Insufficient evidence for recommending the use of zonisamide as a monotherapy AED. Insufficient evidence for recommending the use of zonisamide as an adjunct AED.		

AED(s), anti-epileptic drug(s); BID, bis in die (twice daily); Chloraz, Chlorazepate; CSF, cerebrospinal fluid; CL, confidence level; Diaz, Diazepam; Gaba, Gabapentin; IE, idiopathic epilepsy; LEV, Levetiracetam; m, month(s); NA, Not Available; PB, phenobarbital; PBr, potassium bromide; PO, per os; SF, seizure frequency; SID, semel in die (once daily); TID, ter in die (three times daily); TPM, Topiramate; w, week(s).

**Table 7:** **Details of numbers of dogs, pre- and post- treatment seizure frequency, period of treatment, doses of AED(s), seizure frequency reduction/response after the initiation of treatment and efficacy statements for each study**

**References**	**Schwartz-Porsche et al.** [30]	**Schwartz-Porsche et al.** [15]	**Cunningham et al.** [29]	**Löscher et al.** [26] **Rieck et al.** [27]	**Morton et al.** [31]
**AED evaluated**	**Primidone**				
**2** ^**nd**^ **AED**	-	-	-	-	-
**3** ^**rd**^ **AED**	-	-	-	-	-
**4** ^**th**^ **AED**	-	-	-	-	-
**No of dogs**	30	20	15	26	12
**Period of treatment (months)**	Approx. 6	mean, 14; range, 6.0-35	9	mean, 6.0 ± 0.6	Unclear
**Dose of AED(s) (mg/kg)**	range, 13–100 PO SID	range, 17–107 PO SID	NA	mean, 51; range 24–70 PO SID	median, 50; mean, 48; range, 18–94 PO SID
**Pre-treatment SF (seizures/month)**	NA	NA	NA	mean, 1.75 (over a period of 9 m)	median, 8; mean, 8.5; range, 0–20 (period was not reported)
**Post-treatment SF (seizures/month)**	NA	NA	NA	mean, 0.59	median, 0; mean 0.83; range, 0-8
**No of dogs that were failures**	5/30 (17%)	8/20 (40%)	2/15 (13%)	7/26 (27%)	-
**No of dogs with >0% - <50% reduction in SF**	5/30 (17%)	-	-	-	2/12 (17%)
**No of dogs with ≥50% - <100% reduction in SF**	10/30 (33%)	7/20 (35%)	6/15 (40%)	16/26 (62%)	-
**No of dogs with 100% reduction in SF**	10/30 (33%)	5/20 (25%)	7/15 (47%)	4/26 (15%)	10/12 (83%)
**No of dogs with >30% reduction in SF**	20/30 (67%)	12/20 (60%)	13/15 (87%)	20/26 (77%)	11/12 (92%)
**95% CI successfully treated cases**	50% - 83%	39% - 81%	70% - 100%	61% - 93%	62% - 100%
**Overall evidence for/against recommending the use of an AED**	Insufficient evidence for recommending the use of primidone as a monotherapy AED				

AED(s), anti-epileptic drug(s); BID, bis in die (twice daily); Chloraz, Chlorazepate; CSF, cerebrospinal fluid; CL, confidence level; Diaz, Diazepam; Gaba, Gabapentin; IE, idiopathic epilepsy; LEV, Levetiracetam; m, month(s); NA, Not Available; PB, phenobarbital; PBr, potassium bromide; PO, per os; SF, seizure frequency; SID, semel in die (once daily); TID, ter in die (three times daily); TPM, Topiramate; w, week(s).

**Table 8:** **Details of numbers of dogs, pre- and post- treatment seizure frequency, period of treatment, doses of AED(s), seizure frequency reduction/response after the initiation of treatment and efficacy statements for each study**

**References**	**Nafe et al.** [25]				**Govendir et al.** [21]	**Platt et al.** [22]
**AED evaluated**	**Sodium Valproate**				**Gabapentin**	
**2** ^**nd**^ **AED**	PB (11 dogs)	Primidone (6 dogs)	PB (24 dogs)	-	PB (17 dogs)	PB (11 dogs)
**3** ^**rd**^ **AED**	Phenytoin (11 dogs)	-	-	-	PBr (16 dogs)	PBr (11 dogs)
**4** ^**th**^ **AED**	-	-	-	-	-	-
**No of dogs**	11	6	24	16	17	11
**Period of treatment (months)**	mean, 4.9; range, 1-8				4	3
**Dose of AED(s) (mg/kg)**	range 25–40 PO SID (PB and Phenytoin doses were not reported)	range, 30–45 PO SID; Primidone: NA	range, 30–110 PO SID; PB: NA	range, 25–105 PO SID	median, 35; range, 32–40 PO SID; PB: median, 8; range, 6–12 PO SID; PBr: median, 24; range, 14–30 PO SID	mean, 10.9; 9.3-13.6 PO TID; PB and PBr: NA but were within normal reference values based on the serum levels
**Pre-treatment SF (seizures/month)**	mean, 2.7 (period was not recorded)				median, 2; range, 1–4 (recorded over a period of median 1.5 years)	median, 6; 2–140 (recorded over a period of 3 m)
**Post-treatment SF (seizures/month)**	NA				median, 1; range, 0.5-3	median, 2; range, 0-4
**No of dogs that were failures**	4/11 (36%)	3/6 (50%)	8/24 (34%)	9/16 (56%)	6/17 (35%)	1/11 (9%)
**No of dogs with >0% - <50% reduction in SF**	1/11 (9%)	2/6 (33%)	2/24 (8%)	7/16 (44%)	1/17 (6%)	4/11 (36%)
**No of dogs with ≥50% - <100% reduction in SF**	6/11 (55%)	1/6 (17%)	14/24 (58%)	7/16 (44%)	7/17 (42%)	6/11 (55%)
**No of dogs with 100% reduction in SF**	-	-	-	-	3/17 (17%)	-
**No of dogs with >30% reduction in SF**	6/11 (55%)	1/6 (17%)	14/24 (58%)	7/16 (44%)	10/17 (59%)	9/11 (82%)
**95% CI successfully treated cases**	30% - 84%	−13% - 47%	38% - 78%	20% - 68%	36% - 82%	26% - 84%
**Overall evidence for/against recommending the use of an AED**	Insufficient evidence for recommending the use of sodium valproate either as a monotherapy or an adjunct AED.				Insufficient evidence for recommending the use of gabapentin as an adjunct AED.	

AED(s), anti-epileptic drug(s); BID, bis in die (twice daily); Chloraz, Chlorazepate; CSF, cerebrospinal fluid; CL, confidence level; Diaz, Diazepam; Gaba, Gabapentin; IE, idiopathic epilepsy; LEV, Levetiracetam; m, month(s); NA, Not Available; PB, phenobarbital; PBr, potassium bromide; PO, per os; SF, seizure frequency; SID, semel in die (once daily); TID, ter in die (three times daily); TPM, Topiramate; w, week(s).

**Table 9:** **Details of numbers of dogs, pre- and post- treatment seizure frequency, period of treatment, doses of AED(s), seizure frequency reduction/response after the initiation of treatment and efficacy statements for each study**

**References**	**Dewey et al.** [18]	**Kiviranta et al.** [17]	**Ruehlmann et al.** [35]
**AED evaluated**	**Pregabalin**	**Topiramate**	**Felbamate**
**2** ^**nd**^ **AED**	PB (9 dogs)	PB (10 dogs)	PB (6 dogs)
**3** ^**rd**^ **AED**	PBr (8 dogs)	PBr (8 dogs)	-
**4** ^**th**^ **AED**	-	LEV (1 dogs)	-
**No of dogs**	9	10	6
**Period of treatment (months)**	3	6-15	median, 9
**Dose of AED(s) (mg/kg)**	Pregabalin: 2 PO TID (dose was increased up to until 3–4 PO TID); PB and PBr: NA but were within normal reference values	TPM: 5 PO BID for 2 m, then 10 PO BID for 2 m and then 10 PO TID for 2 m; PB and PBr and LEV: NA but were within normal reference values	Felbamate: median, 63 (initial dose) and 77 (final dose) PO SID; PB: 3.75 PO BID (stopped 2 m after felbamate started)
**Pre-treatment SF (seizures/month)**	median, 4.3; mean, 4.2; range, 2–6.3 (recorded over a period of 3 m)	median, 3.75; range, 2–9 (recorded over a period of 2 m)	median, 3.75 (recorded over a period of median 3.8 m)
**Post-treatment SF (seizures/month)**	median 1.7; mean 1.8; range 0.7-3.3	Range, 4 · 3 ± 2 · 5-4 · 7 ± 5 · 0 (recorded at the 6th m)	2 seizures (in total)
**No of dogs that were failures**	-	3/10 (30%)	-
**No of dogs with >0% - <50% reduction in SF**	2/9 (22%)	2/10 (20%)	-
**No of dogs with ≥50% - <100% reduction in SF**	7/9 (78%)	3/10 (30%)	4/6 (66%)
**No of dogs with 100% reduction in SF**	-	2/10 (20%)	2/6 (34%)
**No of dogs with >30% reduction in SF**	7/9 (78%)	6/10 (60%)	6/6 (100%)
**95% CI successfully treated cases**	51% - 100%	19% - 81%	100%
**Overall evidence for/against recommending the use of an AED**	Insufficient evidence for recommending the use of pregabalin as an adjunct AED.	Insufficient evidence for recommending the use of topiramate as an adjunct AED.	Insufficient evidence for recommending the use of felbamate as an adjunct AED.

AED(s), anti-epileptic drug(s); BID, bis in die (twice daily); Chloraz, Chlorazepate; CSF, cerebrospinal fluid; CL, confidence level; Diaz, Diazepam; Gaba, Gabapentin; IE, idiopathic epilepsy; LEV, Levetiracetam; m, month(s); NA, Not Available; PB, phenobarbital; PBr, potassium bromide; PO, per os; SF, seizure frequency; SID, semel in die (once daily); TID, ter in die (three times daily); TPM, Topiramate; w, week(s).

### Phenobarbital

Seven studies [11,14,15,26,27,31,36] evaluated the efficacy of phenobarbital as a monotherapy agent, giving a combined sample size of 269 dogs. Two studies [11,14] demonstrated low/moderate overall risk of bias, one study demonstrated moderate/high risk of bias [15] and the remaining studies, high overall risk of bias. In all the studies but one [31], the majority of the dogs were treated successfully by oral administration of phenobarbital.

Five studies [11,14,15,26,27] recommended the use of phenobarbital as a monotherapy AED. In two studies [31,36], although the effectiveness of phenobarbital was implied, it was not clearly stated. There was overall good evidence for recommending the use of phenobarbital as a monotherapy AED.

### Imepitoin

Four studies [13,14,26,27] evaluated the efficacy of oral imepitoin either as monotherapy or an adjunct to other AEDs, giving a combined sample size of 278 dogs. Two studies [13,14] demonstrated an overall low/moderate risk of bias and the remaining overall high risk of bias. In one study [14], it was shown that the majority of the dogs were managed successfully with imepitoin. The same study showed non-inferiority of imepitoin compared to phenobarbital.

The studies were in favor of the use of oral imepitoin either as a monotherapy or an adjunct AED to phenobarbital or primidone. There was overall good evidence for recommending the use of imepitoin as monotherapy, but insufficient as adjunct AED.

### Potassium bromide

Seven studies [11,26-28,32-34] evaluated the efficacy of potassium bromide either as monotherapy [11] or as an adjunct to phenobarbital and/or primidone (the remaining studies), giving a combined sample size of 289 dogs. One study [11] demonstrated an overall low/moderate risk of bias, two studies [32,34] demonstrated an overall moderate/high risk of bias and the remaining were classified as at high overall risk of bias. In approximately half of the studies [11,33,34], the majority of the dogs were treated successfully by oral administration of potassium bromide.

All the studies recommended the use of potassium bromide as an AED. One study [11] recommended the use of potassium bromide as a first-line monotherapy AED, although phenobarbital may have been considered more effective as it showed more favorable results compared to potassium bromide. There was overall fair level of evidence for recommending the use of potassium bromide as a monotherapy, but insufficient as adjunct AED.

### Levetiracetam

Three studies [12,16,23] evaluated the efficacy of levetiracetam as an adjunct to other AEDs, giving a combined sample size of 71 dogs. One study [12] demonstrated overall low/moderate risk of bias and the remaining studies overall moderate/high risk of bias. In all the studies, the majority of the dogs were treated successfully by oral co-administration of levetiracetam.

In one study [12], seizure frequency was reduced significantly compared to baseline but no difference was detected when compared to the placebo group (dogs in both the placebo and LEV group were on maintenance therapy with phenobarbital and/or potassium bromide and/or gabapentin). In another study [16], levetiracetam was found to be efficacious initially, but 6/9 responders experienced an increase in seizure frequency after 4–8 months. In the third study, phenobarbital was discontinued in some cases and no increase in seizure frequency was noticed. There was overall fair evidence for recommending the use of levetiracetam as an adjunct AED.

### Zonisamide

Three studies [19,20,24] evaluated the efficacy of oral zonisamide either as monotherapy [24] or as an adjunct to other AEDs (the remaining studies), giving a combined sample size of 33 dogs. The studies demonstrated an overall moderate/high risk of bias with one study [20] classified as at high overall risk of bias. In only one of these studies [20] were the majority of the dogs treated successfully by oral administration of zonisamide.

All three studies recommended the use of oral zonisamide either as a monotherapy or as an adjunct AED to phenobarbital and/or potassium bromide. However, there was overall insufficient evidence for recommending the use of zonisamide either as a monotherapy or as an adjunct AED.

### Primidone

Six studies [15,26,27,29-31] evaluated the efficacy of primidone as a monotherapy agent, giving a combined sample size of 103 dogs. Three studies [15,29,30] demonstrated an overall high moderate/risk of bias and three studies demonstrated an overall high risk of bias [26,27,31]. In all studies but one [15], the majority of the dogs were treated successfully by oral administration of primidone.

All of the studies recommended the use of primidone as a monotherapy AED. In one of these studies [31], primidone was found to be more effective than phenobarbital as a first line monotherapy AED. In another study [15], primidone, although effective, was found to be less preferable as a first-line monotherapy AED compared to phenobarbital due to signs of liver toxicity. There was overall insufficient evidence for recommending the use of primidone as a monotherapy AED.

### Gabapentin

Two studies [21,22] evaluated the efficacy of oral gabapentin as an adjunct to other AEDs, giving a combined sample size of 28 dogs. One study [22] demonstrated an overall moderate/high risk of bias and the other one [21] demonstrated an overall high risk of bias. In none of the studies, there was an increased likelihood that the majority of the dogs were treated successfully by oral administration of gabapentin.

Studies though were in favor of the use of oral gabapentin as an adjunct AED to phenobarbital and potassium bromide but in one of them [22], its use was suggested with reservation. There is currently overall insufficient evidence for recommending the use of gabapentin as an adjunct AED.

### Pregabalin

One study [18] evaluated the efficacy of oral pregabalin as an adjunct to phenobarbital and potassium bromide in 9 dogs. The study demonstrated an overall moderate/high risk of bias. There was an increased likelihood that the majority of the dogs were treated successfully by oral administration of pregabalin. The study supported its use, although there is currently overall insufficient evidence for recommending the use of pregabalin as an adjunct AED.

### Sodium valproate

One study [25] evaluated the efficacy of sodium valproate in different groups either as a monotherapy or as an adjunct to phenobarbital, primidone or a combination of phenobarbital and phenytoin in 57 dogs. The study demonstrated an overall high risk of bias. In this study, there was an increased likelihood that the majority of the dogs were not treated successfully by oral administration of sodium valproate. Although this study stated that sodium valporate could be a useful adjunctive AED, there was overall insufficient evidence for recommending its use.

### Felbamate

One study [35] evaluated the efficacy of felbamate as an adjunct to phenobarbital specifically in dogs with focal IE in 6 dogs. The study demonstrated overall moderate/high risk of bias. All of the dogs (100%) were treated successfully by the oral administration of felbamate. The study supported its use. However, there is currently an overall insufficient evidence for recommending the use of felbamate as an add-on AED.

### Topiramate

One study [17] evaluated the efficacy of topiramate as an adjunct to phenobarbital, potassium bromide and levetiracetam in 10 dogs. The study demonstrated an overall moderate/high risk of bias. In this study, there was an increased likelihood that the majority of the dogs were not treated successfully by the oral administration of topiramate. The study supported its use as a moderately efficient AED. However, there is currently overall insufficient evidence for recommending the use of topiramate as an adjunct AED.

## Discussion

To the authors’ knowledge, this is the first systematic review of AED treatment for canine IE. The authors followed the PRISMA statement to report this systematic review [37]. Twenty-six studies, the vast majority of them UCTs derived from group B, published in two languages were identified and evaluated in this review. In total, 1153 dogs were evaluated. A good level of evidence supported the efficacy of oral phenobarbital and imepitoin as monotherapy AEDs, fair and insufficient level of evidence supported the efficacy of potassium bromide as monotherapy and adjunct AED respectively and fair level of evidence supported the efficacy of levetiracetam as adjunct AED. Favorable results were reported for the efficacy of oral primidone, zonisamide, gabapentin, sodium valproate, pregabalin, felbamate and topiramate as adjunct AEDs, but there was insufficient level of evidence to support their efficacy.

Overall risk of bias ranged from low/moderate to high; only four studies [11-14] categorized as low/moderate overall risk and the remaining as moderate/high or high. Studies in group A which were considered to offer lower overall risk of bias were too few compared to those of group B (study group A:group B proportion was 1:6). Therefore, the results from the studies concerning the efficacy of each AED should be interpreted with caution. In addition, only 17% and 10% of the 29 studies included well characterized groups and evaluated good numbers of dogs, respectively. None of the bRCTs included well characterized groups and only two of them evaluated a good number of dogs. The same studies, though, were considered to offer the highest quality of evidence among all the studies and recommended the use of phenobarbital and imepitoin in particular as well as potassium bromide and levetiracetam as AEDs. However, mainly due to the small number of bRCTs and to a lesser extend due to the inadequate disease definitions and study group sizes, definitive suggestions concerning their efficacy are precluded.

**Figure 1:**
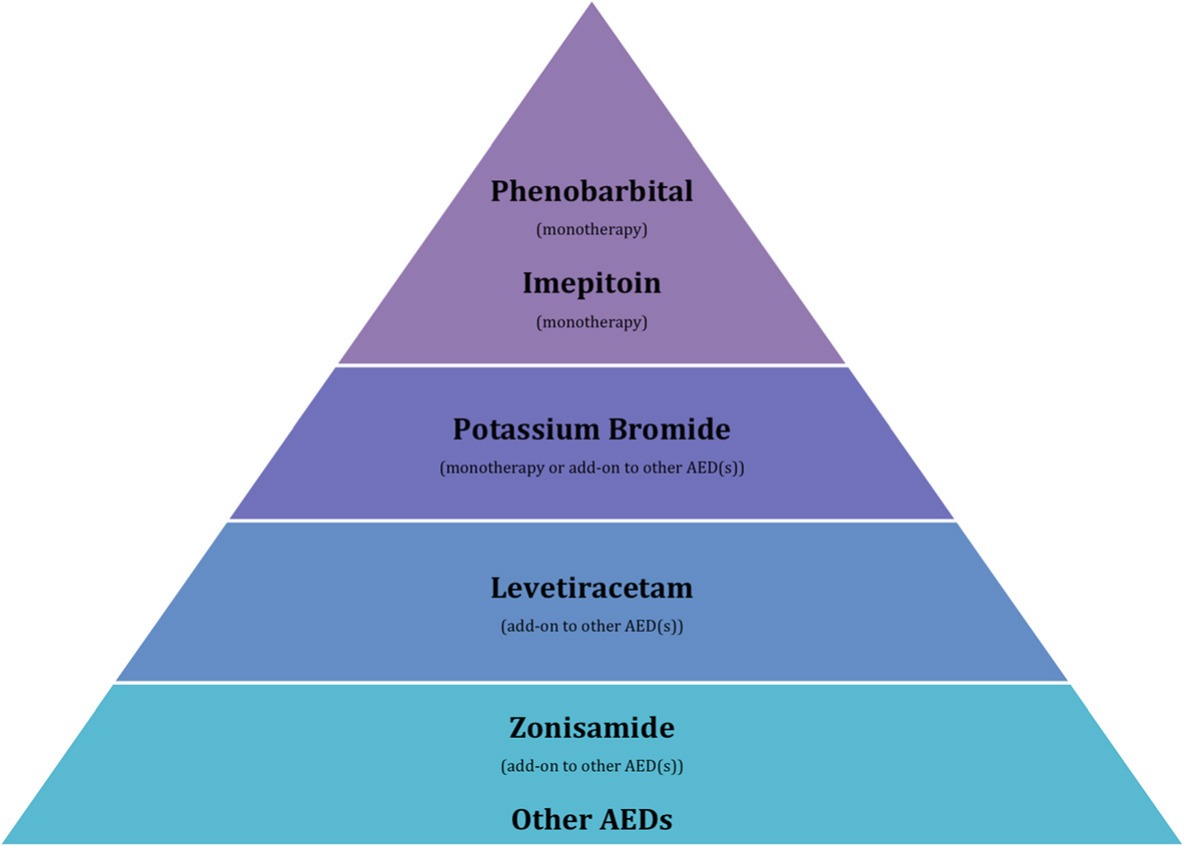
**Pyramid of hierarchy describing the recommendation of AEDs based on the assessment of their efficacy and quality of evidence.**

Based on the level of quality of evidence provided by studies for each AED as well as the assessment of their efficacy, a pyramid of hierarchy was proposed (Figure 1). Phenobarbital and imepitoin were found to be at the top of the pyramid. In human epilepsy, many AEDs are used for the management of seizures but phenobarbital remains one of the most important; meta-analyses of RCTs found that only minor differences occur on the grounds of efficacy between phenobarbital and other established AEDs [38]. Although phenobarbital may remain the most efficient AED in human epilepsy, its tolerability issues lead to the investigation and use of other AEDs with almost the same efficacy but more tolerable. Imepitoin was initially developed as a new AED for humans, but development was ceased because of differences in pharmacokinetic values between smokers and non-smokers, although the tolerability of this drug in humans was high [39].

In canine epilepsy there are limitations in treatment of IE due to the rapid elimination of the majority of the AEDs with only few, i.e. phenobarbital, primidone and potassium bromide, having sufficient half-life [40,41]. The same drugs have been approved for treatment of canine epilepsy in Europe and/or USA, with phenobarbital to be one of the most effective and well-known AED. Recently, imepitoin was also approved for the treatment of canine epilepsy based on some RCTs [13,14]. Monotherapy with imepitoin in dogs with newly diagnosed epilepsy showed that it was moderately less effective but potentially more tolerated than phenobarbital or primidone. Also, in dogs with chronic epilepsy receiving phenobarbital and/or primidone, most dogs exhibited a reduction in seizure frequency and severity after adjunctive therapy with imepitoin [26,27]. In a laboratory study, imepitoin was compared with phenobarbital in an acute canine seizure model using pentylenetetrazole, resulting in a comparable anticonvulsant efficacy [42]. In the European pseudo-placebo trial, high dose (30 mg/kg PO BID) of imepitoin was compared to low dose (1 mg/kg PO/BID) and results showed that seizure frequency was significantly reduced in the first compared to the second group [13]. In the same study, baseline seizure frequency was different between the two groups; thus, the change in seizure frequency reduction between the groups was significant. In a US field study, imepitoin was compared to primidone but failed to demonstrate non-inferiority to primidone [13]. In the European pivotal multicenter bRCT, imepitoin was given as monotherapy and compared to phenobarbital in dogs with newly diagnosed epilepsy [14]. The study suggested that imepitoin seems to be similarly effective to and more well-tolerated than phenobarbital and suggests that there is no need for serum levels measurements as indicated for phenobarbital. However, despite blinding a greater number of dogs dropped out and were excluded from analysis in imepitoin compared to phenobarbital group and the different results in the analysis pre- and post-exclusion was questioned to have potentially biased the results in favor of imepitoin [13]. On the other hand, although imepitoin allows quick titration and dose adjustments in dogs with poor seizure control, this was not followed in the pivotal study due to the comparison with phenobarbital which demands slow titration and this could have disadvantaged imepitoin [14]. Lastly, there are currently no placebo or pseudo-placebo controlled trials for phenobarbital as for imepitoin, which only suggests how efficient phenobarbital is compared to other AEDs (i.e. potassium bromide or imepitoin) but not to a true negative control group. Based on the above, we could state that imepitoin might be as efficient as phenobarbital. The conduction of further bRCTs for both drugs may be needed to confirm this.

Based on the results and outcomes of this systematic review, phenobarbital and imepitoin could be used as “first line” medications in the cascade for the management of IE. However, individual practices and clinicians can follow another cascades depending on the drugs licensed in their countries.

Several aspects of the review process may have adversely affected the selection and assessment of the reviewed studies as well as the response to treatment. First of all, factors associated with dogs’ signalment (i.e. breed, age, sex) may have influenced the response to treatment. Various breeds are represented in all the studies reporting baseline characteristics, but none of the studies, apart from one [36], reports outcomes specific to breed.

In addition, the origins of the cases were different in some studies. All studies enrolled cases from referral centers, of which three [21,31,34] also enrolled cases from first-opinion centers. Studies recruiting dogs only from a referral center are at risk of enrolling cases of ‘more difficult to treat’ or drug resistant epilepsy. Studies recruiting dogs from first-opinion centers could enroll cases that have been diagnosed, treated or managed with different protocols compared to referral cases, which makes the results slightly heterogeneous.

The range of study publication dates may have influenced the reliability of the diagnosis, as diagnostic methods (e.g. brain MRI) have improved since the earliest publication. It has been reported, however [4,43], that based only on the history, signalment and clinical examination, the diagnosis is likely to be IE and that further diagnostic investigation is not always needed. However, it could be argued that advanced brain imaging and diagnostic techniques were not well developed or commonly used when these papers were published. A more recent study reports a low likelihood of revealing an underlying lesion by MRI, in seizuring dogs <6 years of age with an unremarkable interictal neurological examination [44]. Significant variability in the diagnostic process between studies may have led to heterogeneity of the type of epilepsy enrolled in the drug trials, potentially influencing the response of dogs to the AEDs. Diagnostic procedures have varied between the 26 studies from only a report of the history, signalment and neurological examination as the main criteria for diagnosing IE to performance of brain MRI or CT and/or CSF analysis for confirmation. Almost all of the studies included in this review based a diagnosis of IE on signalment, history and neurological examination. Many of the studies also reported the use of advanced brain imaging and other diagnostic techniques to confirm the diagnosis, however, based on the criteria set out in the review protocol, only four of the studies were considered to include well-characterized cases with an advanced work-up such as MRI and CSF analysis.

Several factors either clinical or related to the natural history of the disease have been reported to influence the outcome of treatment with AEDs in several studies. Heterogeneity in treatment initiation and protocols between studies has been observed throughout this review. These differences are potential sources of variability in the recorded treatment response. Indeed, in recent rodent studies, it was reported that early treatment initiation affected positively the likelihood of remission of specific types of epilepsy [45]. In human epilepsy, patients are treated with AEDs sooner in an attempt to increase the chances of remission. However, there is evidence that remission rates in countries with and without immediate AED treatment initiation are similar, demonstrating that AEDs do not affect the likelihood of remission in all types of epilepsy, but suppress the seizures [46]. In addition, the variability in AED combinations between the studies of this review and the fact that AEDs’ administration was stopped at variable durations of treatment could highly influence the response to treatment. Also, there is further evidence from many studies, that other aspects of disease such as high seizure frequency before treatment and occurrence of status epilepticus or cluster seizures can affect the response to drug and consequently the treatment outcome [36,47-51]. In one of the studies [32], most of the canine population (90%) was presented with cluster seizures, before potassium bromide was added, which could have affected the outcome.

The 29 studies evaluated did not report outcomes based on a common, standardized set of outcome measures. Although all of the included studies reported outcome measures for each AED based on the proportion of dogs which had 0% (or increased), <50%, ≥50% or 100% reduction in seizure frequency, marked heterogeneity was evident among studies (i.e. variations among the studies in treatment duration required to achieve clinical success, time point at which treatment success was assessed and rates of relapse after dose alterations). Therefore, it was difficult to make sensible comparisons of measures of treatment efficacy and duration across multiple studies, even when the efficacy of the same AED was studied.

It was not possible to retrieve further information about patients and their therapy other than that reported in the publications, which may have improved the assessment of each of the criteria for assessing the bias. According to PlosOne policy, all the data of a study should remain available to the readers [52]. One example where the bias could have been introduced in this systematic review is by using different types of publications. Data were sometimes easier and sometimes less easy to extract, e.g. in the case of EMEA report [13]. Furthermore, trial protocols were not sought to aid the assessment of selective reporting. Occurrence of blinding was clearly reported in only three studies. In the rest, lack of blinding was detected based on the methods. Randomization of treatment groups was described in only four studies. In addition, most of the studies included in this review were pre/post intervention and retrospective case series studies, which did not compare the results with a control group. Consequently, the reported efficacy of the AED examined is likely to be exaggerated.

Also, many studies on epilepsy were designed to evaluate for a positive response to therapy, which is defined as a ≥50% reduction in seizures [6]. In one study which combines the results from three placebo-controlled trials, a large proportion of dogs with IE responded to placebo alone, with an approximately 30% reduction in seizures following placebo administration [53]. However, the dogs in the placebo group were on conventional AED therapy that could have affected the results and outcome. This placebo response has been attributed to the regression to the mean effect, the natural waxing and waning course of epilepsy over time, the likelihood for improved patient care during participation in the trial, and investigator and participant bias [53]. The presence of the strong placebo effect found in that study suggests that results from non-blinded studies, particularly those that involve a small number of animals and short follow up time should be interpreted with some caution. Based on this finding, a calculation in dogs derived from our included nbRCT, uncontrolled CTs and case series was performed to report the proportion of dogs which had >30% reduction in seizure frequency (details in Tables 3, 4, 5, 6, 7, 8 and 9). Generally, a range of 57%-84% (phenobarbital), 60%-92% (primidone), 65%-75% (imepitoin), 32%-100% (potassium bromide), 60%-92% (levetiracetam), 62.5%-100% (zonisamide), 17%-58% (sodium valproate), 59%-82% (gabapentin) as well as 78% (pregabalin), 100% (felbamate) and 60% (topiramate) cases were reported to have >30% reduction in seizure frequency.

Due to the tendency in publications to report positive outcomes, the exclusion of unpublished and full-length studies (e.g. conference abstracts) could bias the results (publication bias). Also, study funding or support was declared in eight studies and all of them reported favorable efficacy of the evaluated drug.

Meta-analytic approach was not possible due to the within-study variations in baseline characteristics of the dogs involved (such as age, breed and gender), the significant differences between study designs (such as the evaluation of different AEDs, inclusion of studies with a quite different design) and the several potential sources of bias that were identified.

## Conclusion

Oral phenobarbital and imepitoin in particularly as well as potassium bromide and levetiracetam are likely to be effective in the treatment of canine IE. Only four bRCT [11-14] were reported. The same studies were considered to offer the highest quality of evidence amongst all the studies evaluated. The efficacy of phenobarbital, imepitoin, potassium bromide and levetiracetam have all been supported by at least one bRCT, but no bRCTs were identified for the remaining AEDs. This fact, in combination with the variations in baseline characteristics of the dogs involved, the significant differences between study designs and several potential sources of bias that were identified preclude definitive recommendations. Therefore, this systematic review underlines the need for greater numbers of adequately sized, blinded, randomized controlled trials evaluating the efficacy of all the AEDs (specifically for those that bRCTs are not available) for IE.

## Abbreviations

AED(s)1: Anti-epileptic drug(s)
BID: Bis in die (twice daily)
bRCTs: Blinded randomized clinical trials
Chloraz: Chlorazepate
CSF: Cerebrospinal fluid
CL: Confidence level
CT: Computer tomography
CTs: Clinical trials
Diaz: Diazepam
Gaba: Gabapentin
IE: Idiopathic epilepsy
LEV: Levetiracetam
m: month(s)
MRI: Magnetic resonance imaging
NA: Not available
nbRCTs: Non-blinded randomized clinical trials
NRCTs: Non-randomized clinical trials
PB: Phenobarbital
PBr: Potassium bromide
PO: Per os
SF: Seizure frequency
SID: Semel in die (once daily)
TID: Ter in die (three times daily)
TPM: Topiramate
w: week(s)
